# The Microscope in Medicine

**Published:** 1891-02-14

**Authors:** Frank J. Wethered


### The Microscope in Medicine.

By Frank J. Wethered, M.D.

### V.—STAINING SECTIONS.

After the sections have been cut they may be kept in small bottles containing equal parts of methylated spirits and water. Each bottle should be carefully labelled with the date and nature of the specimens therein contained. It is sometimes advisable to examine the sections unstained, but as a general rule, some colouring matter is used in order to bring out more clearly the minutiæ of structure. The chief exceptions to this rule are those objects which possess sufficient colour, owing to the presence of pigment, which characteristic would be lost by the employment of artificial dyes. Examples of this are brown induration of the lung or small capillary aneurisms in the brain. The larger parasites, as, for example, the trichina spiralis, are best mounted unstained.

The variety of stains now in use are very numerous, and are constantly being added to. It would not only be undesirable, but useless to attempt to describe here all the colouring matters in use. The majority of them are only used in bacteriological work, and these will be referred to when describing the processes of staining and identifying the different pathogenic microbes. Other stains, again, are employed on account of the chemical reaction which they produce in the tissues. Such are iodine green, methyl violet, and safranin, etc., for demonstrating amyloid material; ferrocyanide of potassium, as used for showing the presence of iron in the liver in cases of pernicious anæmia, and also the modern methods of staining the central nervous system introduced by Weigert and Pal.

For ordinary purposes an intimate knowledge of two methods of staining sections is sufficient. Undoubtedly the two best are (1) Hæmatoxylin and Eosin, (2) Lithium Carmine and Picric acid. The former is suitable for all specimens, but more especially for the demonstration of cirrhotic processes. It has the disadvantage of not being permanent, and is therefore best adapted for use in diagnosis. Carmine is also suitable for any structures, with the exception of those in which it is intended to demonstrate fatty changes. This stain is very permanent, and may therefore be employed for sections which it is found desirable to preserve. It is rather trying to the eyes, and does not show up well by artificial light.

*Hæmatoxylin and Eosin.*—In staining by this process, if the sections have been previously placed in alcohol, they must be removed for a couple of hours to distilled water before being placed in the stain. The best formula is that introduced by Ehrlich, which is as follows:—Hæmatoxylin, 2 parts; Alcohol, 100 parts; Distilled Water, 100 parts; Glycerine, 100 parts; Alum, 2 parts; Acetic Acid, 5 parts.

The solution is not ready for use until eight days after it has been prepared.

In another article the manipulation of specimens will be described. It is sufficient to say here that the staining is best conducted in small glass capsules, and that these should be kept covered by ground glass covers in order to prevent the entrance of dust; and for neat and successful work it is absolutely essential that all stains should be filtered previous to use. The sections are most conveniently removed from one receptacle to another by means of needles, and should be kept spread out as flat as possible, otherwise they may become stained unequally. For staining in Hæmatoxylin two methods are adopted; in the first the sections are merely dipped in the undilute stain and then at once washed in distilled water.

In the second method, which is decidedly preferable, the original stain is diluted with distilled water, about six drops of the former being added to a watch-glassful of the latter, the mixture being filtered before use. Sections are allowed to remain in it for about ten minutes, and are then removed to a capsule of distilled water. If insufficiently stained they are replaced in the Hæmatoxylin for a minute or two; experience alone can teach when the right tint has been reached. The effect of the stain is greatly enhanced by allowing the sections to float in water some hours. The sections are next counter-stained in eosin. The solution best suited for the purpose has the following composition: Eosin, 5 parts; distilled water, 100 parts. Specimens are allowed to soak in this dye for about five minutes, and are then washed in water previous to dehydration and mounting, as will be described later on.

Stained in this manner, the nuclei of the cells will be coloured a deep purple, whilst the ground substance will have assumed a clear rose colour which particularly picks out the fibrous tissue. A caution may here be given as regards the ease with which the eosin is washed out by the absolute alchohol used for dehydration, and this process therefore must be carried out as rapidly as possible.

*Carmine and Picric Acid.*—Staining with these two dyes may be conducted in one process by the solution known as picro-lithium-carmine, but it is more satisfactorily managed if the stains are used separately. The carmine solution has the following composition:—Saturated Solution of Lithium Carbonate, 100 parts; Carmine, 2·5 parts. To be digested and filtered.

The specimens are placed in the undiluted stain for ten minutes and are then thoroughly washed in acidulated alcohol until no more colour can be extracted. The "acidulated alcohol" is prepared according to the following formula:—Hydrochloric Acid, 1 part; Alcohol, 70 parts; Distilled Water, 30 parts.

The counter stain is a saturated alcoholic solution of picric acid. A few drops of this are added to a watch-glassfull of alcohol, and the sections are allowed to remain in it for about five minutes. The same caution is here necessary as with eosin, as the yellow colour is very quickly removed by absolute alcohol; in fact, it is a good plan to add a couple of drops of the picric acid to the absolute alcohol used for dehydrating. In sections thus stained the nuclei are picked out very clearly, and in a brilliant red colour, whilst the remainder of the tissue is stained yellow. After mounting, some of the yellow dye is often diffused through the balsam, but this need give rise to no uneasiness, as it is speedily reabsorbed. After being stained by either of the above methods the sections are dehydrated, cleared, and mounted, as will be described in the next article.

